# Blunt Injury Forearm Debridement Under Ultrasound-Guided Regional Anesthesia for a Marfan Syndrome Patient

**DOI:** 10.7759/cureus.12729

**Published:** 2021-01-15

**Authors:** Vamsi krishna Uppalapati, Nikhilesh Kundu, Deb Sanjay Nag, Rajiv Shukla

**Affiliations:** 1 Anesthesiology, Tata Main Hospital, Jamshedpur, IND; 2 General Surgery, Tata Main Hospital, Jamshedpur, IND

**Keywords:** marfan syndrome, aortic dilatation, wound debridement, regional anesthesia, ultra sound guided brachial plexus nerve block

## Abstract

Marfan syndrome is an autosomal dominant connective tissue disorder with anomalies involving the musculoskeletal system, cardiovascular system, skin, eyes, and teeth. Patients with Marfan syndrome are especially prone to cardiovascular complications, which increases the risk multifold under general anesthesia. This is a case of a 37-year-old Marfan syndrome male patient with cardiac manifestations and his anesthesia course during emergency wound debridement.

## Introduction

Marfan syndrome is an autosomal dominant hereditary connective tissue disease with an estimated prevalence of 1/5000 newborns, of which 25 to 30% are new mutations. Marfan syndrome impacts multiple systems like skeletal, ocular, and cardiovascular. Aortic dilation and dissection are the primary causes for mortality and morbidity in this group of patients [1], however, other organs such as the skin, palate, lungs, and dura can also be affected. The most significant manifestation in Marfan syndrome is the gradual dilation of the aortic root and ascending aorta, leading to aortic valve incompetence and aortic dissection [2]. The anesthetic management of Marfan syndrome patients is challenging and the literature on the treatment of these cases is sparse. Neuraxial anesthesia in Marfan syndrome will result in sudden hemodynamic changes and collapse. Marfan syndrome is frequently associated with dural ectasia and continues cerebrospinal fluid (CSF) leakage into epidural or subdural space leading to CSF hypovolemia and inadequate neuraxial block. However, with ultrasound-guided in-plane supraclavicular brachial plexus nerve block, hemodynamics becomes more stable [3]. The current case of a 37-year-old male presents high risk towards general anesthesia. After thorough cardiovascular evaluation, ultrasound-guided in-plane supra clavicular brachial plexus nerve block was the choice of anesthesia management [4]. 

## Case presentation

Marfan syndrome is an inherited condition that affects several systems and structures. The clinical presentation and nature of the disorder vary from person to person, age, and gender. The symptomatic variations are also observed within the same family structure. Many individuals with Marfan syndrome may not have any of the characteristics and/or symptoms identified with Marfan syndrome. In certain instances, this disorder will not be recognized by the parents and may be asymptomatic [5]. In this case, the patient presented all the external physical conditions along with cardiovascular abnormalities like severe aortic regurgitation, global hypokinesia, and low ejection fraction of 20%. Old pulmonary Koch's and severe kyphoscoliosis were additional risk factors.

Upon general inspection, the patient was taller and had a slim build, the lower portion of his body being stretched with respect to the upper half. He was 89 kg in weight and 6.4 inches in height [6]. His arms, thighs, and thumbs were elongated. The patient had a positive wrist (Walker-Murdoch sign: a distal phalanx of first and last fingers of the hand overlapping along the wrist) and a positive thumb (Steinberg sign: a flexed thumb caught in a clenched fist protrudes past the ulnar boundary of that hand).

**Figure 1:**
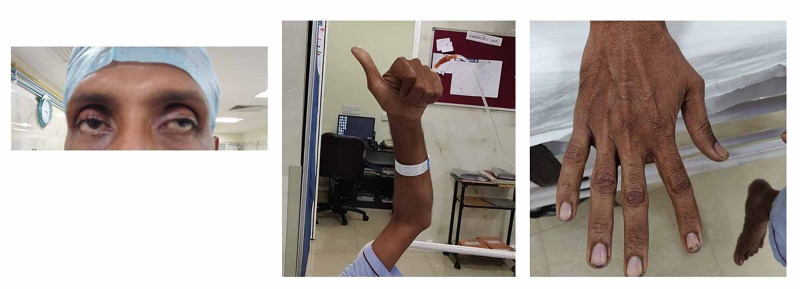
V Shaped Eyes, Wrist, Fingers

The ocular conditions associated with our patient like V pattern strabismus, enophthalmos, and high myopia were observed as shown in Figure 1.

**Figure 2:**
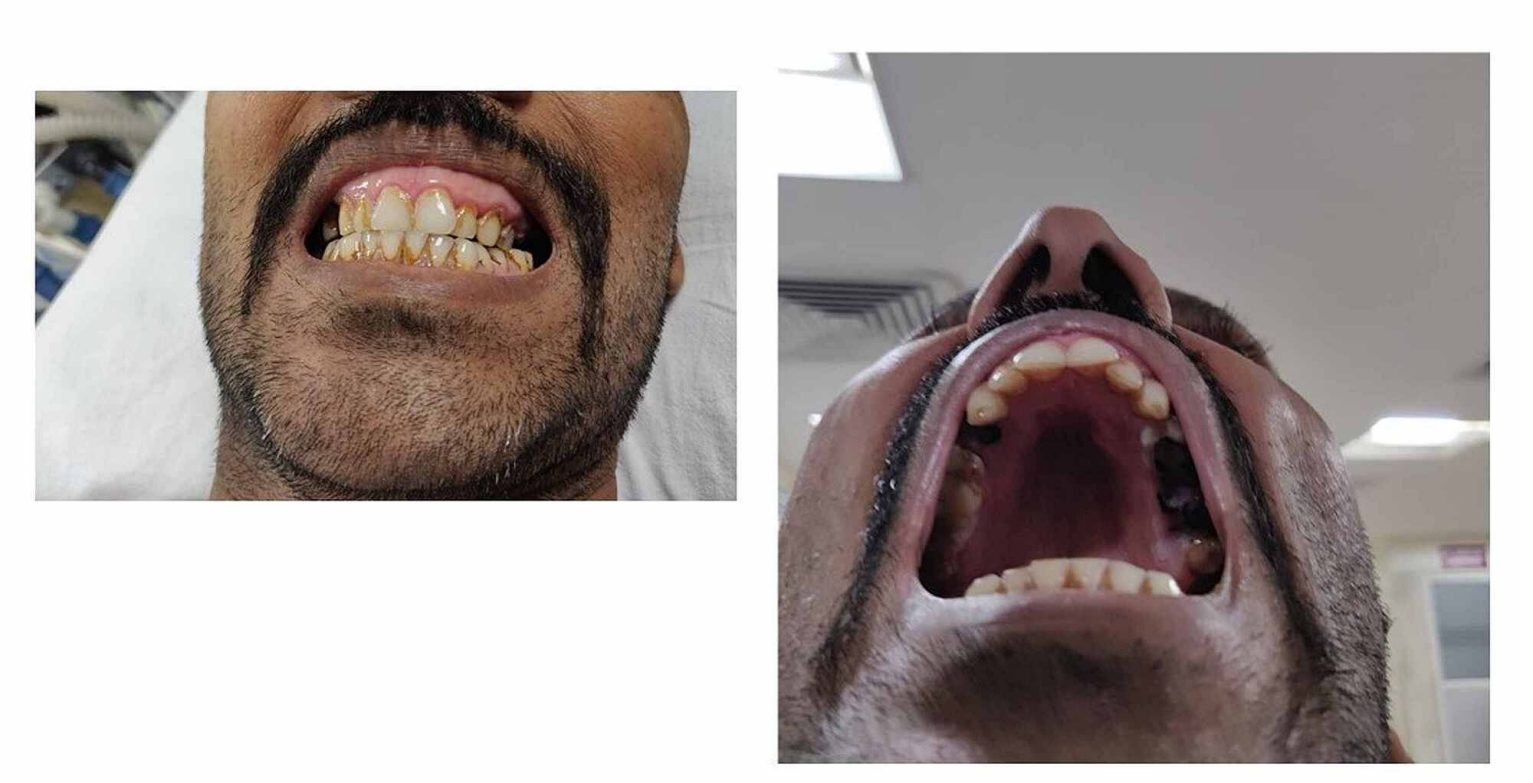
Dentals

In addition, orthodontic care was needed and suggested to the patient because of his high arched palate and dental crowding, shifted midline along with open crossbite, which are also classic conditions seen in patients with Marfan syndrome, which makes him prone for an anticipated difficult airway [7]. Hence avoided general anesthesia. Figure 2 shows the developed defects of the teeth, of which the most prominent are the supernumerary teeth; enamel defects, dentinogenesis imperfecta, dental dysplasia, and jaw cyst formation have been identified. Patient came with a forearm blunt wound.

## Discussion

Our Marfan syndrome patient presented with a set of multiple cardiovascular complications which would be at very high risk when put under general anesthesia. Hence his forearm wound debridement surgery was then decided to be carried out by choosing ultrasound-guided in-plane supraclavicular brachial plexus nerve block [8,9].

Besides systemic complications, Marfan syndrome impacts the personality and quality of life of the affected person [10]. The weakened physical ability and cosmetic problems restrict the life prospects for patients, as well as contribute to dissatisfaction and poor self-esteem. Maxilla constriction, crowded dentures, and concomitant cross-bites are typical of oral manifestations for which patients undergo a cosmetic correction. Such cases often display high-arch palate combined with narrow nasal airways and compensated mouth breathing. The patient is prone to obstructive sleep apnea which makes them prone for anticipated difficult airway [11].

Due to high mortality and morbidity associated with aortic dissection, anesthetic management of patients with Marfan syndrome is a difficult situation [12]. We explain the anesthetic treatment of a patient with Marfan syndrome who needed emergency forearm wound debridement surgery under ultrasound-guided in-plane supraclavicular brachial plexus nerve block, location right forearm, duration of surgery 45 mins, administration of 10 ml of 2% xylocaine + 10 ml of 0.25% bupivacaine following ultrasound-guided supra clavicular nerve block in-plane technique single pocket.

## Conclusions

Marfan syndrome is one of the most prevalent hereditary connective tissue disorders and this case is considered attributable to a wide spectrum of coronary, musculoskeletal, and vascular anomalies arising in a single patient. With a blunt wound trauma in his forearm, wound debridement under ultrasound-guided in-plane supraclavicular brachial plexus nerve block could minimize fatal complications and makes it unique for discussion.

## References

[1] Meijboom L (2005). Cardiovascular Complications in Patients With the Marfan Syndrome. https://dare.uva.nl/search?identifier=599e9a92-4882-400d-a213-23a4da37bda9.

[2] Attenhofer Jost CH, Greutmann M, Connolly HM (2014). Medical treatment of aortic aneurysms in marfan syndrome and other heritable conditions. Curr Cardiol Rev.

[3] Baghizada L, Krings T, Carvalho JC (2012). Regional anesthesia in Marfan syndrome, not all dural ectasias are the same: a report of two cases. Can J Anesth.

[4] Gauss A, Tugtekin I, Georgieff M, Dinse‐Lamb A, Keipke D, Gorsewski G (2014). Incidence of clinically symptomatic pneumothorax in ultrasound‐guided Infraclavicular and supraclavicular brachial plexus block. Anesthesia.

[5] Mckusick VA (1995). Heritable disorders of connective tissues. III. The Marfan syndrome. J Chronic Dis.

[6] Nwosu BU, Lee MM (2008). Evaluation of short and tall stature in children. Am Fam Physician.

[7] Bostanci BE, Korkut E, Unlu N (2017). Dental findings in marfan syndrome: a case report. J Istanb Univ Fac Dent.

[8] Egmond PW, Schipper IB, van Luijt PA (2012). Displaced distal forearm fractures in children with an indication for reduction under general anesthesia should be percutaneously fixated. Eur J Orthop Surg Traumatol.

[9] Araujo MR, Marques C, Freitas S, Santa-Bárbara R, Alves J, Xavier C (2016). Marfan Syndrome: new diagnostic criteria, same anesthesia care?. Case Rep Rev.

[10] Naidoo P, Ranjith N, Zikalala Z, Mahoney S, Ho K (2018). Marfan syndrome: a case report and pictorial essay. Pan Afr Med J.

[11] Jain E, Pandey RK (2013). Marfan syndrome. BMJ Case Rep.

[12] Ghatak T, Samanta S, Samanta S (2013). Anesthetic management of a patient with Marfan syndrome and severe aortic root dilatation undergoing cholecytectomy and partical hepatic resection. Saudi J Anaesth.

